# The First Oral Fixed-Dose Combination of Netupitant and Palonosetron for the Treatment of Chemotherapy-Induced Nausea and Vomiting

**DOI:** 10.6004/jadpro.2016.7.1.5

**Published:** 2016-01-01

**Authors:** Joseph W. Coyne

**Affiliations:** Coyne Consulting, Mundelein, Illinois

The world is witnessing a continuous rise in the incidence of various types of cancer, which results in an increasing number of patients undergoing chemotherapy. According to the online cancer database GLOBOCAN by the International Agency for Research on Cancer (IARC), globally 14.1 million people were diagnosed with cancer (excluding nonmelanoma skin cancer) in 2012, compared with 12.7 million in 2008 (Research and Markets, 2014).

The incidence rate is projected to reach 15.2 and 17.1 million in 2015 and 2020, respectively. These findings indicate that the growing incidence of various types of cancer is likely to induce oncologists to prescribe chemotherapy to their patients for the effective treatment of cancer, thereby driving the chemotherapy-induced nausea and vomiting (CINV) drugs market. Generally, 25% to 30% of patients with cancer receive chemotherapy as a treatment option, and 70% to 80% of these patients undergoing chemotherapy may exhibit nausea and vomiting as major symptoms (Research and Markets, 2014).

Considered one of the most distressing side effects of chemotherapy (Decker, DeMeyer, & Kisko, 2006), CINV continues to have a great impact on the quality of life of patients receiving certain antineoplastic therapies (Cohen, de Moor, Eisenberg, Ming, & Hu, 2007). It can be classified as acute, delayed, or anticipatory.

Acute CINV occurs within 24 hours of chemotherapy administration (Rice, 2011). Delayed CINV presents more than 24 hours for up to several days following administration of chemotherapy. Anticipatory CINV occurs within 12 hours prior to the scheduled treatment administration and has been reported to in up to 25% of patients (Sorrell-Camp, 2005). A study conducted by Glaus et al. (2004) reported that delayed nausea had a negative impact on performance status (PS) and activities of daily living (ADLs), as measured by the Functional Living Index–Emesis (FLIE) scale in 75% of patients receiving moderately to highly emetogenic chemotherapy.

## INDICATION

Netupitant/palonosetron (Akynzeo) is an oral fixed-dose combination of netupitant, a substance P/neurokinin 1 (NK1) receptor antagonist, and palonosetron, a serotonin-3 (5-HT₃) receptor antagonist indicated for the prevention of acute and delayed nausea and vomiting associated with initial and repeat courses of cancer chemotherapy, including, but not limited to, highly emetogenic chemotherapy. Oral palonosetron prevents nausea and vomiting during the acute phase, and netupitant prevents nausea and vomiting during both the acute and delayed phases after cancer chemotherapy (Eisai, 2015).

## MECHANISM OF ACTION AND DOSING

The development of acute emesis is known to depend on serotonin; specifically, the 5-HT3 receptors have been demonstrated to selectively stimulate the emetic response. Delayed emesis has been largely associated with the activation of the tachykinin family NK1 receptors substance P. As shown in in vitro and in vivo studies, netupitant inhibits substance P–mediated responses (Eisai, 2015).

One capsule of netupitant/palonosetron (300 mg of netupitant/0.5 mg of palonosteron) is administered orally approximately 1 hour prior to the start of chemotherapy. It can be taken with or without food (Eisai, 2015).

## CLINICAL STUDIES

As noted, one of the components of netupitant/palonosetron is oral palonosetron, which was approved in 2008 for prevention of acute CINV in moderately emetegenic chemotherapy (MEC), but is not available in the United States.

There are two noninferiority studies with oral and intravenous (IV) palonosetron. One study in patients with MEC demonstrated equivalency in acute CINV, which led to the US Food and Drug Administration (FDA) approval, and the acute highly emetegenic chemotherapy (HEC) study (Study 4) demonstrated comparable effectiveness between oral and IV palonosetron.

Study 4 was a multicenter, multinational, randomized, active-controlled, double-blind, double-dummy, parallel-group, clinical noninferiority study. It was intended to confirm the efficacy and safety of oral palonosetron at 0.5 mg in the prevention of nausea and vomiting induced by HEC in comparison to IV palonosetron at 0.25 mg, focusing on the acute phase. A total of 739 patients (oral palonosetron n = 370; IV palonosetron n = 369) received study medication (Eisai, 2015).

The primary efficacy endpoint was complete response (CR, defined as no emetic episode and no use of rescue medications) within 24 hours (acute phase) after the start of cisplatin-based chemotherapy administration.

In the oral palonosetron arm, 89.4% of patients achieved a CR in the acute phase compared with 86.2% of patients in the IV palonosetron arm, with a difference of 3.21% (99% confidence interval [CI] = –2.74% to 9.17%). Noninferiority of oral palonosetron vs. IV palonosetron was demonstrated, since the limits of the two-sided 99% CI of the difference in proportions was greater than the predefined noninferiority margin set –15%.

In addition to the noninferiority study, there were two efficacy studies performed: the first an HEC study with a primary endpoint of CR during the overall phase (0–120 hours) and the second a doxorubicin/cyclophosphamide study in which its primary endpoint was CR in the delayed phase (25–120 hours; Eisai, 2015).

Study 1 was conducted by Hesketh et al. (2014), in patients who were receiving HEC. This trial was a multicenter, randomized, parallel, double-blind, phase II dose-ranging study. A total of 135 patients were randomized to the netupitant/palonosetron arm, and all patients received a cisplatin-based chemotherapy regimen, either as monotherapy or with concomitant chemotherapy.

In the primary endpoint of CR during the overall phase (0–120 hours), netupitant/palonosetron demonstrated a significantly greater CR rate of 89.6% compared with 76.5% for oral palonosetron (p = .003). Netupitant/palonosetron also demonstrated significant CR rates in the acute (0–24 hours) and delayed (25–120 hours) phases, including a 98.5% CR rate in the acute phase (Hesketh et al., 2014).

Study 2 was a trial by Aapro et al. (2014) in patients who were receiving AC chemotherapy. This study was a multicenter, multinational, randomized, double-blind, double-dummy, parallel-group, phase III superiority study. A total of 1,455 patients were randomized to receive netupitant/palonosetron plus dexamethasone or oral palonosetron at 0.5 mg plus oral dexamethasone. The primary endpoint of this study was CR during the delayed phase (25–120 hours). This study also included a multiple-cycle extension phase for patients who completed cycle 1.

In the primary endpoint of this study, netupitant/palonosetron demonstrated a significantly greater CR rate of 76.9%, compared with 69.5% for oral palonosetron (*p* = .001). In the same study, patients receiving AC had the option of enrolling in a multiple-cycle extension for up to seven additional cycles of chemotherapy. The results from cycles 2–6 in the delayed phase (see Figure) are presented with 95% confidence (Aapro et al., 2014).

**Figure:**
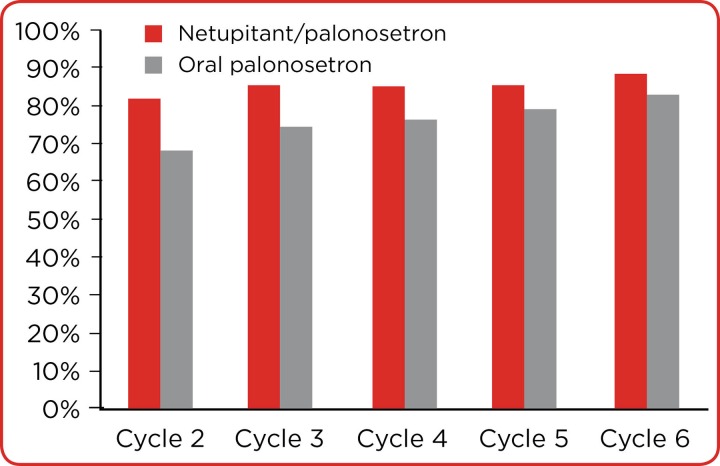
Proportion of patients with a complete response in the delayed phase by treatment group and cycle in Study 2. Adapted from Eisai (2015).

A phase III safety study conducted by Gralla et al. (2014), with a primary endpoint to assess safety (see Safety section), also evaluated the efficacy of netupitant/palonosetron over multiple cycles of HEC and MEC. A total of 413 chemotherapy-naive patients received netupitant/palonosetron in a 3:1 randomization over standard of care. Patients completed 1,961 total chemotherapy cycles (76% MEC, 24% HEC), with 75% completing more than four cycles. The overall (0–120 hours) CR rates in cycle 1 were 81% for netupitant/palonosetron and 76% for aprepitant and palonosteron; antiemetic efficacy was maintained over repeated cycles (Gralla et al., 2014).

## SAFETY

The most frequent netupitant/palonosetron related adverse events included constipation (3.6%) and headache (1.0%). The majority of adverse events reported were mild to moderate. Electrocardiographic (ECG) changes, including QT interval prolongation, have been seen in patients receiving other 5-HT₃s. In addition, torsade de pointes (an abnormal heart rhythm) has been reported in some patients receiving other 5-HT₃s.

The FDA issued a safety warning based on these changes, recommending ECG monitoring in patients with electrolyte abnormalities (e.g., hypokalemia or hypomagnesemia), congestive heart failure, bradyarrhythmia, or those taking concomitant medications that prolong the QT interval (FDA, 2012). No cardiac safety concerns based on ECGs were reported with netupitant/palonosetron in the findings of the phase III safety study conducted by Gralla et al. (2014).

## GUIDELINES

Currently, both the National Comprehensive Cancer Network (NCCN) and the American Society of Clinical Oncology (ASCO) recommended a combination of 5-HT₃ and NK1 agents for the management of MEC- and HEC-associated CINV. See the Table for a summary of these recommendations from NCCN and ASCO.

**Table:**
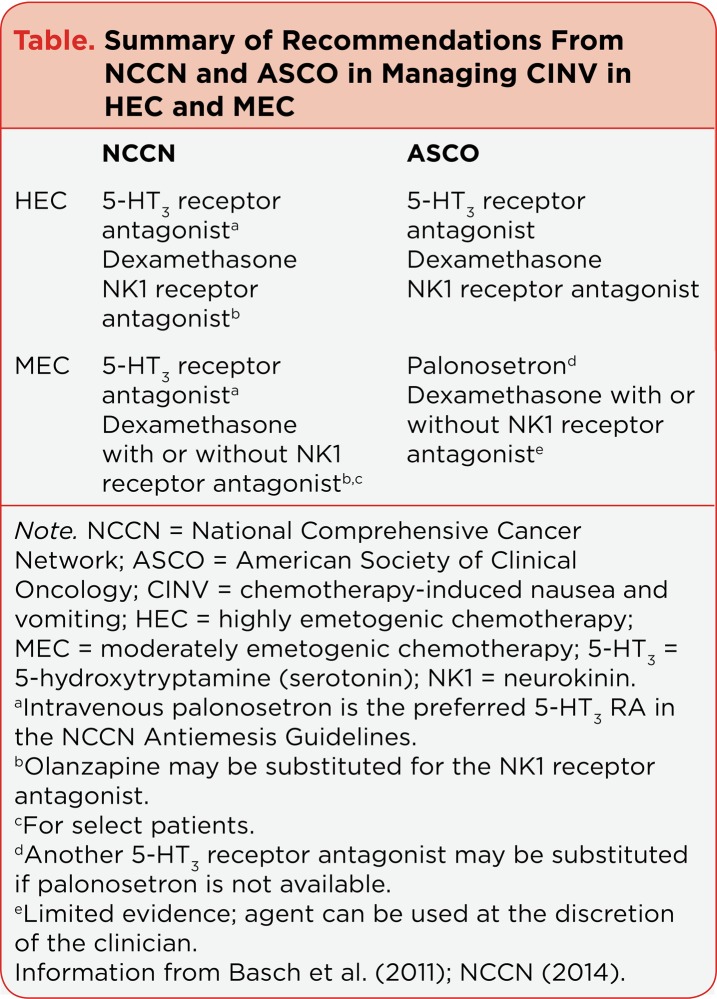
Summary of Recommendations From NCCN and ASCO in Managing CINV in HEC and MEC

## ROLE IN THERAPY

With so many choices currently in the 5-HT₃ and NK1 therapeutic areas, where does netupitant/palonosetron fit in therapy? It clearly meets both NCCN and ASCO guidelines for the management of MEC- and HEC-induced nausea and vomiting and therefore should be used in practice. Additionally, netupitant/palonosetron’s indication does not currently exclude its use in low emetogenic chemotherapy.

Considering the compliance issues after treatment and the possible need for rescue medication in some patients, netupitant/palonosetron can be given to these patients to simplify what is most likely an already complex medication regimen. Many of the current medications provide coverage for either acute or delayed CINV, necessitating multiple drug therapy for proper control. The administration of the single fixed-dose capsule eliminates the need for individual 5-HT₃ and NK1 medications, and its CR rate for acute and delayed nausea and vomiting eliminates the need for rescue medications, which may contribute to an overall improved quality of life.

Netupitant/palonosetron may present its own unique challenge by requiring the patient to fill the prescription at a retail pharmacy and then remember to take it prior to treatment. As it is an oral medication, some providers require the use of the prescription drug benefit opposed to the medical benefit used for IV medications necessitating this change. Thus, Medicare patients would pay a portion of the fee for an in-clinic antiemetic, but could pay 100% if they are in the donut hole for prescription drug coverage. Physicians with in-office dispensing capabilities may provide an alternative to the retail pharmacy for some patients; however, many patients will need to navigate this new facet of chemotherapy premedication.

Netupitant/palonosetron will reduce infusion chair time by eliminating the need for two separate IV medications (a 5-HT₃ and an NK1), which has traditionally been a common practice with both agents being offered in an oral dosage form. An additional benefit of this reduced chair time is the practice’s ability to alleviate what is most likely an already overcrowded infusion center with long patient wait times.

## COST OF THERAPY

In the current health-care climate and a time of increasing financial demands, a new medication evaluation would not be complete without some mention of cost. The CINV space has so many possible combinations encompassing multiple medication classes that it makes a simple comparison difficult.

Each provider and practice should consider both direct and indirect costs in his or her review. The direct and indirect costs of CINV are also significant. These costs include those related to the acquisition of antiemetic drugs as well as expenses associated with unscheduled office or emergency department visits, hospitalization admissions, and loss of productivity for patients and their caregivers (Haiderali, 2011). Individual agent cost is important, as is the need for breakthrough medication and its costs (both direct and indirect).

Questions to consider follow. Does medication adherence with breakthrough medication require after-hour calls, days lost at work, decreased quality of life, and potential delays in other components of treatment? Does your practice dispense breakthrough medications as part of an in-office dispensing model? If so, what are your costs to process multiple prescriptions, not only for initial management of CINV but for breakthrough/rescue symptom management? What about inventory management costs? Would it benefit your practice to inventory a single agent for the management of CINV as opposed to multiple agents? Would this step contribute to the potential decrease in medication errors related to the timing of administration?

Another consideration in treatment selection is the financial impact on patients. Currently, netupitant/palonosetron has the ability to be reimbursed on both medical and prescription benefits. Each of these reimbursement paths presents its own financial effect on patients. The prescription benefit could result in high out-of-pocket expenses for those not qualifying for copay assistance, as is the case with government payers.

All of these as well as other questions should be addressed by each practice based on each individual situation, patient population, and practice model, so an informed decision can be made surrounding the use of netupitant/palonosetron and its potential cost implications for the provider’s practice as well as for his or her patients. Regardless of the financial implications, the clinical data clearly suggest that netupitant/palonosetron may have a place in the management of CINV.

## SUMMARY

Chemotherapy-induced nausea and vomiting continues to be a significant issue for patients with cancer and has been shown to negatively impact quality of life. Although both the NCCN and ASCO agree on treatment guidelines for emesis control in these patients, the prevalence of CINV remains high.

The INSPIRE study found that for patients receiving HEC regimens and an antiemetic regimen consistent with NCCN guidelines, 0.8% experienced CINV compared with 62.2% of patients whose regimen was inconsistent with guidelines (Gilmore et al., 2014). As netupitant/palonosetron is the first oral fixed-dose combination of a 5-HT₃ (palonosetron) and an NK1 (netupitant) and has demonstrated both clinical efficacy and safety in CINV prevention in both MEC and HEC regimens, it provides a convenient way to follow guidelines, improve issues of patient adherence, and minimize the potential need for rescue medications.
